# Debris Removal Using a Hydroxyapatite Nanoparticle-Containing Solution (Vector Polish) with Sonic or Ultrasonic Agitation

**DOI:** 10.3390/ma14164750

**Published:** 2021-08-23

**Authors:** Michael Hülsmann, Christoph Beckmann, Steffi Baxter

**Affiliations:** 1 Department of Conservative and Preventive Dentistry, Center for Dentistry, University Zurich, Plattenstrasse 11, CH 8032 Zurich, Switzerland; michael.huelsmann@med.uni-goettingen.de; 2 Department of Oral and Maxillofacial Surgery, University of Aachen, 52074 Aachen, Germany; chrbeckmann@ukaachen.de; 3 Department of Preventive Dentistry, Periodontology and Cariology, University of Göttingen, 37075 Göttingen, Germany

**Keywords:** hydroxyapatite nanoparticles, dentinal debris, grooves, passive ultrasonic irrigation, sonic irrigation

## Abstract

Chemomechanical preparation of the root canal system is considered to be the most important part of root canal treatment, including both mechanical removal of tissue remnants and dentine chips, and chemical elimination of biofilm and microorganisms. A number of different solutions and agitation techniques have been proposed for that purpose. It was the aim of the present study to investigate whether root canal cleanliness can be improved by using a hydroxyapatite nanoparticle-containing solution with and without sonic or ultrasonic agitation. Seventy-four single-rooted teeth were divided into four experimental groups (*n* = 15) and two control groups (*n* = 7). All teeth were split longitudinally and a groove and three holes were cut into the root canal wall and filled with dentinal debris. Final irrigation was performed using sodium hypochlorite or a hydroxyapatite nanoparticle-containing solution (Vector polish) activated with a sonically or an ultrasonically driven endodontic file. Two calibrated investigators rated the remaining debris using a four-score scale. The results were analyzed using a non-parametric test with α < 0.05. Sonic and ultrasonic irrigation with sodium hypochlorite cleaned the grooves and holes well from debris. The hydroxyapatite nanoparticles activated by a sonic file cleaned grooves and holes equally well. Ultrasonically activated nanoparticles performance was clearly inferior. The syringe control-group left large amounts of debris in grooves and holes. The use of the hydroxyapatite nanoparticles used in this study did not improve removal of debris.

## 1. Introduction

Chemomechanical preparation of the root canal system is considered to be the most important part of root canal treatment, including both mechanical removal of tissue remnants and dentine chips, and chemical elimination of biofilm and microorganisms. A plethora of materials, solutions and techniques have been used for cleaning and disinfection of root canals but so far no irrigant nor any technique have been identified as being equally effective in both regards [1]. Among chemical disinfectants sodium hypochlorite in various concentrations is still recommended as the irrigant of choice due to its good antimicrobial properties, despite its limited capability to dissolve organic tissue, to remove the biofilm and to penetrate into the intricacies of the complex root canal system.

In order to improve the mechanical efficacy of root canal irrigation agitation of the irrigant is recommended. In the 1950s, ultrasonics was introduced for endodontic purposes, which later—among others—was modified by Cunningham and Martin [2], Weber et al. [3], and van der Sluis et al. [4], now termed as Passive Ultrasonic Irrigation (PUI). More recently, sonically driven agitation using stainless steel or NiTi metal wires or even plastic tips (e.g., EndoActivator (Dentsply Maillefer, Ballaigues, Switzerland), EDDY (VDW, Munich, Germany)) has become popular among endodontists [5].

Nanoparticles have recently gained considerable attention in medicine and dentistry (“nanodentistry”), and also in endodontics, mainly due to their antimicrobial properties [6,7,8,9,10,11,12]. Their main characteristic is an extremely small particle size. At least 50% of particles in such a solution must be sized between 1–100 nm to be classified as a nanomaterial. In addition, nanoparticles show a large ratio between mass and surface area, and a high chemical reactivity [13].

In endodontics, different kinds of nanoparticles (e.g., gold, silver, copper, zinc, titanium, chitosan, calcium oxide, calcium hydroxide, and hydroxyapatite) have been proposed as irrigants, as medical gels to be in direct contact with infected dentine, as antibacterial ingredients to endodontic sealers, or as media to be activated by lasers in photodynamic therapy (PDT). Due to their positive electrical charge nanoparticles are capable to disturb the activity of cell membranes thus interfering with the metabolism of bacteria [13,14,15], and also exerting a strong antimicrobial effect [16,17]. Shrestha et al. [8] demonstrated that nanoparticles agitated by ultrasonics can be transported by collapsing microbubbles into dentinal tubules at a depth of up to 1000 µm. Combining good antimicrobial efficacy and ultrasonically driven intense mechanical action on the root canal walls nanoparticles could be a suited solution for irrigation of root canals. No information is available so far on the combined effect of nanoparticles and sonically driven agitation.

The ultrasonic system Vector (Dürr Dental, Bietigheim-Bissingen, Germany) is used in periodontal treatment for removal of subgingival calculus without causing destruction to the root cementum [15]. Vector polish, a solution containing synthetically produced hydroxyapatite nanoparticles with <10 µm size, is activated by an ultrasonically driven instrument at approx. 25 kHz and 30 µm amplitude [18,19]. Braun et al. [19] supposed that the ultrasonic energy is transferred directly to the root through the nanoparticles.

SEM studies demonstrated that the use of Vector polish fluid in combination with application of the Vector ultrasonic system results in well cleaned and smooth external root surfaces with well-retained dental hard tissue [20]. In endodontics, nanoparticles to the best of our knowledge so far have been investigated only with regard to their antibacterial properties but no study was found investigating the mechanical action of a solution containing hydroxyapatite nanoparticles in terms of removal of tissue remnants and dentinal debris from root canals when activated by sonic or ultrasonic devices.

Therefore, the aim of this study was to investigate the ability of the Vector polish hydroxyapatite nanoparticle solution as a root canal irrigant activated by passive ultrasonic agitation (PUI) or sonic agitation (SI) and irrigation using a common syringe (needle irrigation, NI) as a control to remove dentinal debris from grooves and holes inside straight root canals.

The nill-hypothesis was that there is no difference in the cleaning ability between sodium hypochlorite and a hydroxyapatite nanoparticle-containing solution when agitated by sonic or ultrasonic devices.

## 2. Materials and Methods

Seventy-four single rooted teeth without previous endodontic treatment and with mature roots were selected and cleaned with hand scalers (HLW-Germany, Wernberg-Köblitz Germany). All teeth were shortened to 19 mm length and the access cavities were prepared using diamond burs (Brasseler, Lemgo, Germany). The root canal orifices were flared with Gates-Glidden burs sizes 2 and 3 (Brasseler). The insertion depth of the Gates-Glidden burs was limited to 6 mm from the incisal edge. The root canals were prepared with the Mtwo rotary NiTi-system (VDW, Munich, Germany) to a size of 40/0.04. Following each instrument size, the root canals were rinsed with 2 mL NaOCl (3%) (Hedinger, Stuttgart, Germany). The final flush was performed using 5 mL ethylenediaminetetraacetic acid EDTA (17%) (Lege artis, Dettenhausen, Germany) and 5 mL NaOCl (3%). The irrigants were delivered using a syringe with a 30-gauge needle.

Silicone moulds were made of Silaplast (Detax, Ettlingen, Germany) and pushed in acryl tubes in order to mount the teeth in a reproducible position for standardized preparation and irrigation.

**Figure 1:**
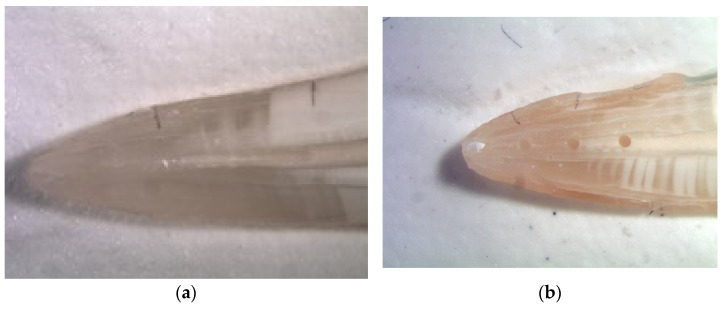
(**a**) Groove prepared into one half of the split root; (**b**) Three holes prepared into the other half of the split root.

Following preparation two longitudinal grooves were sliced into the root with a diamond disk (Horico, Berlin, Germany) without exposing the root canal and the root was split into two halves with a small chisel. Only root halves that could be reassembled perfectly were included in the further experiments. As suggested by Lee et al. [21] in one of the root halves a standardized groove (4 mm long, 0.5 mm deep, and 0.2 mm wide) was cut into the dentin using modified finger spreaders ISO-size 35 (VDW). Three holes were cut into the opposite root halves using round burs (Brasseler, Lemgo, Germany) with a diameter of 0.3 mm and with a distance of 2 mm between the holes. The position of the grooves and holes were the same as described by Lee et al. [21] (Figure 1a,b). The root halves were placed in flat silicone beds in order to allow standardized photography in a reproducible position. Photographs were taken from the empty grooves and holes which then were filled with dentine debris under a microscope. The dentine debris had been scraped off from moist root canal walls from teeth not used in this study. The debris was filled into the holes and grooves using a spoon excavator and a small spatula and slightly compressed with blunt gutta-percha cones to achieve a dense packing without voids. Again, photographs were taken of the filled artificial grooves and holes and the root halves were reassembled in the silicone moulds. 

The nanoparticles used in this study were synthesized hydroxyapatites (Vector polish, Dürr, Bietigheim-Bissingen, Germany) commonly used in periodontal treatment for cleaning and root smoothening. The nanoparticles are available in two sizes, namely 25 µm and smaller than 10 µm, the latter solution was used in this study. 

After filling the grooves and holes with dentinal debris and reassembling the root halves in the silicone mould the specimens were randomly divided into six groups: Two groups with 30 teeth each for comparison of the nanoparticle solution (Vector polish, Dürr) and sodium hypochlorite. The 30 samples of each group were divided into two subgroups of 15 teeth each to compare sonic and ultrasonic agitation of both solutions. Two groups with seven teeth each served as control groups (no irrigation, syringe irrigation (NI). 

The ultrasonic agitation (PUI) was performed using the Irrisafe ISO 20 ultrasonic tip (Acteon, Mettmann, Germany), the sonic agitation (SI) used the Komet SF65 tip (Brasseler, Lemgo, Germany), a flexible NiTi irrigation tip. In the control group syringe irrigation was performed with a 30-gauge needle (Vedefar, Dilbeek, Belgium) mounted on a 5 mL syringe (Braun, Melsungen, Germany). 

Three activation cycles with 10 mL 3% sodium hypochlorite or 10 mL Vector polish were performed with the sonic or ultrasonic tips for 20 s. The syringe group was limited to a volume of 10 mL of the irrigants in three parts lasting 20 s each. 

**Figure 2:**
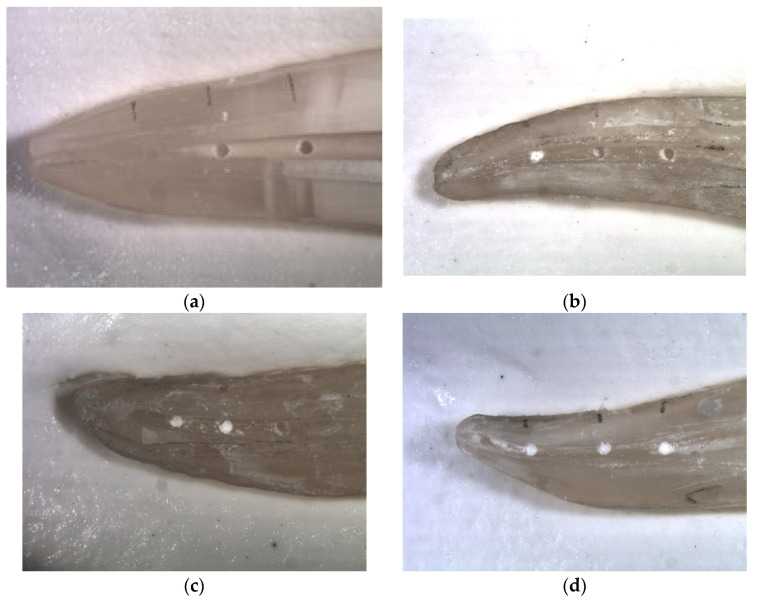
(**a**) Score 0: 0–25% of the holes filled with debris; (**b**) Score 1: 26–50% of the holes filled with debris; (**c**) Score 2: 51–75% of the holes filled with debris; (**d**) Score 3: 76–100% of the holes filled with debris.

**Figure 3:**
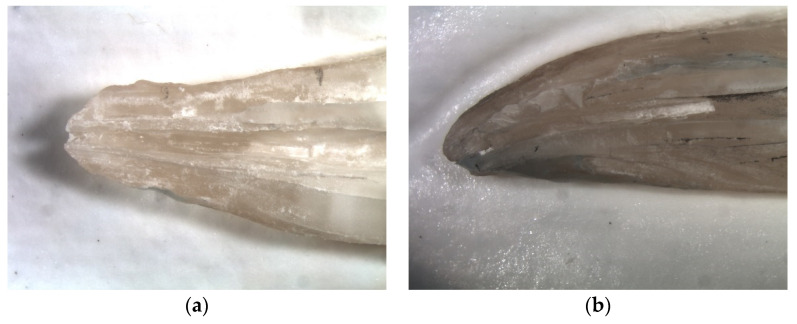

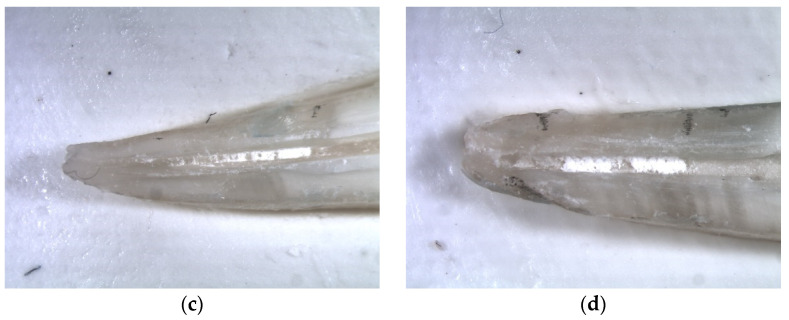
(**a**): Score 0: 0–25% of the groove filled with debris; (**b**) Score 1: 26–50% of the groove filled with debris; (**c**): Score 2: 51–75% of the groove filled with debris; (**d**) Score 3: 76–100 of the groove filled with debris.

After irrigation the root halves were separated again, and final photographs were taken. Each root half was photographed three times: following preparation with empty cavities, cavities filled with dentine debris, and following final irrigation. The photos of the rinsed root halves were independently evaluated by two investigators using a four-score scale:

(a) Score 0: 0–25% of the grooves or holes filled with debris (Figure 2a and Figure 3a)
(b) Score 1: 26–50% of the grooves or holes filled with debris (Figure 2b and Figure 3b)
(c) Score 2: 51–75% of the grooves or holes filled with debris (Figure 2c and Figure 3c)
(d) Score 3: 76–100% of the grooves or holes filled with debris (Figure 2d and Figure 3d).

Before the two investigators performed the final rating 40 photographs representing all scores were rated independently by both investigators for calibration.

## 3. Statistical Evaluation

Statistical evaluation of the results was performed using the Mann–Whitney-U-Test and a non-parametric test with α < 0.05. Intraobserver reproducibility and interobserver agreement were evaluated using Cohen’s Kappa.

## 4. Results

Cohen’s Kappa coefficient for interobserver agreement was 0.85, demonstrating very good interobserver agreement. Cohen’s Kappa coefficient for intraobserver reproducibility was 0.95 for the first and 0.92 for the second observer, respectively, also demonstrating very good intraobserver agreement. There was no statistically significant difference between grooves and holes regarding cleanliness (Mann–Whitney-U-Test, *p* = 0.09), therefore the results for both were pooled and not analyzed separately.

The results of the final scoring and the levels of statistical significance are summarized in Table 1. For PUI and SI activation of sodium hypochlorite the score 0 was reached in 98.3% or 100% of the samples, respectively. The nanoparticle solution failed to remove the dentine debris when using PUI for activation. Sonic activation of the nanoparticle solution was able to remove the dentine debris in 85% of the samples. The syringe irrigation (NI) failed to clean the grooves and holes using either nanoparticles or sodium hypochlorite (Table 1).

The results of the statistical analysis are summarized in Table 2.

## 5. Discussion

The design of the present study is similar to that proposed by Lee et al. [21] and van der Sluis et al. [22] and has frequently been used for investigation of removal of dentine debris [23,24] or calcium hydroxide [23] from root canals. Following longitudinal splitting the root halves can be reassembled using a silicon mould which is unlikely to result in disturbances in the irrigant’s hydrodynamics. This design allows to prepare standardized anatomical features such as holes and grooves which can be filled with dentine chips under close direct visual control. Whereas these holes and grooves cannot exactly reproduce anatomical irregularities inside root canals such as lateral extensions, undercuts, internal resorption lacunae or anastomoses, they allow to create identical conditions in all teeth, assuring a high degree of standardization. 

Lee et al. [21] and van der Sluis et al. [22] used similar scoring systems, allowing reliable and reproducible scoring of the remaining debris. Interobserver agreement and intraobserver reproducibility in the present study showed good results with Kappa-values > 8.85. 

The hydroxyapatite nanoparticle suspension Vector polish so far has not been used or investigated for endodontic purposes although a good cleaning ability has been demonstrated in studies on cleaning root surfaces [15,20]. Unfortunately, no data are available on the concentration of the particles in this solution. It cannot be excluded that different concentrations of the solution would influence the hydrodynamics and energy transfer of the activated solution and consequently also have an impact on the cleaning efficacy. 

No statistically significant differences were found between the post-irrigation appearance of grooves and holes, which is in agreement with the findings of Lee et al. [21]. Rödig et al. [23] using an identical study design reported significantly superior cleanliness of the grooves. The author supposed that this might be related to the larger surface of the grooves which could be addressed better by the hydrodynamical effects of the activated irrigant.

Regarding the removal of debris from root canals no significant difference between sonic or ultrasonic activation of the irrigant or syringe irrigation has been observed in some studies [25,26]. This is in accordance with the results of this investigation for irrigation with sodium hypochlorite. No explanation for the differing behavior of the nanoparticle solution with significantly better results for the sonic agitation could be found in the literature. Interestingly, Kanter et al. [27] also reported a significantly superior performance of a sonic device (EndoActivator) compared to PUI in the removal of debris from prepared canine teeth, which was confirmed by Mancini et al. [28]. The overall results in that study were clearly inferior to those of the present study. Paragliola et al. [29] demonstrated a deeper penetration of dentinal tubules with PUI than with the sonically driven EndoActivator. Conversely, Jensen et al. [30] and Arslan et al. [31] did not find any significant difference between both activation systems. Gu et al. [32], in a review on contemporary irrigant activation techniques, concluded that the results of sonic and ultrasonic techniques and devices are completely inconclusive and dependent on a large number of variables, which is confirmed by the present study. The results in the study of Arslan et al. [31], who compared debris removal from two apical grooves, again were clearly worse than those of the present study. It remains to be investigated whether and to which degree time of activation and intensity of sonic or ultrasonic agitation of the solution influence the degree of cleanliness. 

In the present study nanoparticles showed an inferior cleaning ability than sodium hypochlorite only when agitated with ultrasonics. It can be speculated that the microbubbles created by ultrasonics are too small to transfer enough energy to the nanoparticles, which in consequence are not actively pushed into the grooves and holes. Ohl et al. [33] in a series of experiments demonstrated that high intensity ultrasound is able to press chitosan nanoparticles (size approx. 100 nm) into dentinal tubules. In this regard ultrasonics performed significantly better than sonic agitation using the EndoActivator [34]. It is well known that ultrasonics induces formation of microbubbles which generate shock waves when collapsing and also cause microstreaming with shear stress on the root canal wall [35]. This shear stress not only can disrupt biofilms but also can loosen and remove debris and smear layer [36]. It still has to be elucidated whether the hydroxyapatite nanoparticles used in this study prevent creation of these phenomena or even disturb the effect of shock waves and shear stress on the dentinal wall and which influence the size of the particles has on the energy transfer and the cleaning effiacy. 

Finally, it should be noted that the viscosity of nanoparticles is clearly higher than that of sodium hypochlorite. Cavitation may be generated even in highly viscous emulsions, nevertheless this phenomenon may be dampened by the nanoparticles. Whether electrokinetic transport can improve the distribution and effectiveness of hydroxyapatite nanoparticles, as already demonstrated for chitosan nanoparticles [17] still has to be elucidated.

The nill-hypothesis that there is no difference between the irrigation techniques could be accepted only in parts.

## 6. Conclusions

Using hydroxyapatite nanoparticles did not result in sufficient removal of dentinal debris from grooves and holes inside a root canal when activated with ultrasound. When activating the solution with a sonic tip clean grooves and holes were achieved as well as by activation of sodium hypochlorite with sonics or ultrasound. The hydroxyapatite nanoparticle solution used in this study did not improve root canal cleanliness when compared to sodium hypochlorite.

## Figures and Tables

**Table 1:** Absolute and relative frequencies of added scores for holes and grooves (max. 60, for syringe irrigation max. 28).

Group	Score 0 n/%	Score 1 n/%	Score 2 n/%	Score 3 n/%
SI + NaOCl	60 100%	0	0	0
SI + nanoparticles	51 85%	6 10%	3 5%	0
PUI + NaOCl	59 93.3%	1 6.7%	0	0
PUI + nanoparticles	17 28.3%	3 5%	12 20%	28 46.7%
Syringe irrigation (NI) + NaOCl	10 35.7%	0	8 28.6%	10 35.7%
Syringe irrigation (NI) + nanoparticles	1 3.6%	2 7.1%	5 17.9%	20 71.4%

**Table 2:** Results of the statistical test (level of significance *p* ≤ 0.05). Significant differences in bold.

Group	Groups	*p*
NaOCl	SI vs. PUI	0.876
nanoparticles	SI vs. PUI	**0.000**
SI	NaOCl vs. nanoparticles	0.157
PUI	NaOCl vs. nanoparticles	**0.000**
syringe irrigation (NI)	NaOCl vs. nanoparticles	**0.008**

## Data Availability

Not applicable.
